# Cannabidiol improves haloperidol-induced motor dysfunction in zebrafish: a comparative study with a dopamine activating drug

**DOI:** 10.1186/s42238-023-00177-w

**Published:** 2023-03-04

**Authors:** Akihiro Hasumi, Hideyuki Maeda

**Affiliations:** Department of Forensic Medicine, Tokyo Medical University, 6-1-1 Shinjuku Shinjuku-ku, Tokyo, 160-8402 Japan

**Keywords:** Cannabinoid, Antipsychotic, Parkinsonism, D2 receptor, Light and dark zones

## Abstract

**Background:**

Cannabidiol (CBD) extracted from the cannabis plant is believed to have a medicinal value due to its neuroprotective effect via anti-inflammatory and antioxidant action. Recent behavioral studies in rats have reported that CBD mediates serotonin (5-HT1A) receptor action to improve motor dysfunction induced by dopamine (D2) receptor blockade. In particular, its effect on D2 receptor blockade in the striatum is an important function associated with neurological disorders resulting from various extrapyramidal motor dysfunctions. Dopaminergic neurodegeneration associated with this site is known for inducing Parkinson’s disease (PD), which often affects the elderly. It is also known to cause drug-induced Parkinsonism. This study examines the ameliorating effect of CBD, which does not act directly on D2 receptors, against drug-induced motor dysfunction induced by the antipsychotic drug (haloperidol).

**Methods:**

We created a drug-induced Parkinsonism model in zebrafish larvae using an antipsychotic drug (haloperidol). We evaluated the distance traveled and repetitive light-stimulation response. Furthermore, we examined whether administration of several concentrations of CBD ameliorates symptoms of the Parkinsonism model and compared its effects with those of antiparkinsonian drug ropinirole.

**Results:**

CBD concentrations equal to half of haloperidol’s resulted in an almost complete reversal of haloperidol-induced motor dysfunction, as measured by the distance traveled by the zebrafish and their response to light-stimulus. While ropinirole also significantly reversed haloperidol’s effects at the same concentration as CBD, CBD was more effective than ropinirole.

**Conclusions:**

CBD-induced motor dysfunction improvement via D2 receptor blockade is a potential novel mechanism for the treatment of haloperidol-induced motor dysfunction.

## Background

Cannabinoids are chemical components extracted from cannabis plants. They are widely used globally as luxury goods, similar to cigarettes and alcohol, and as medicines, and their various pharmacological effects have been elucidated in previous researches (Gamelin et al. 2020; Oomen et al. 2018). Cannabidiol (CBD), unlike delta-9-tetrahydrocannabinol (THC), has relatively low psychological and addictive effects (Gamelin et al. 2020) and exhibits anti-inflammatory and antioxidant effects on nerve cells. Owing to its protective ability, research on the efficacy of CBD administration as an epilepsy treatment is in progress (Devinsky et al. 2017; Ferreira-Junior et al. 2020; Jose et al. 2018; Cascio and Pertwee 2014). However, many factors remain unclear because the potential effects of cannabinoids on neurotransmission and pharmacological effects on motor function have not been fully elucidated (Campos et al. 2016). Nevertheless, understanding the cannabinoid system in zebrafish could help establish a basis for investigating the effects of cannabinoids in humans (Bailone et al. 2022).

Our previous study revealed various factors affecting the mortality rate and toxicity associated with the use of synthetic cannabinoids (Hasumi et al. 2020). A significant result was the finding that administration of CBD improves motor function to its original state when motor dysfunction is induced by the synthetic cannabinoid WIN55,212-2 R-(+)-[2,3-Dihydro-5-methyl-3-[(4-morpholinyl)methyl]pyrrolo[1,2.3-de]-1,4-benzoxazin-6-yl]-(1-naphthalenyl)methanone monomethanesulfonate). WIN55,212-2, which showed the same pharmacological action as THC in a previous study (Lawston et al. 2000), induces more behavioral abnormalities than CBD, is highly toxic, and can even cause sudden death upon intake. Understanding the inhibitory effect of CBD against WIN55,212-2 may help unravel CBD’s molecular function. Furthermore, CBD activates the serotonin 1A receptor (or 5-HT1A receptor) (Espejo-Porras et al. 2013), which is known to improve motor dysfunction due to striatal dopamine (D2) receptor inhibition induced by haloperidol to create catalepsy symptoms (Andreza et al. 2016; Gomes et al. 2013). The striatal D2 receptor in the basal ganglia is an important protein associated with Parkinson’s disease (PD) (Patricio et al. 2020).

PD is an extrapyramidal disorder causing involuntary movements, such as those observed in Huntington’s disease, catalepsy, and dyskinesia (Ferreira-Junior et al. 2020; Khisti et al. 1997; Taherianfard et al. 2020; Schmidt and Beninger 2006; Jamwal and Kumar 2019). PD is common among the elderly worldwide and is a serious progressive neurodegenerative disease (Mhyre et al. 2012). A syndrome that causes symptoms similar to PD is called Parkinsonism (Hayes 2019). It has various causes; however, inhibition of dopamine neurotransmission is implicated (Bay Kønig et al. 2019). Antipsychotic drugs used in the treatment of schizophrenia, such as haloperidol (HAL), block dopamine receptors and cause drug-induced Parkinsonism as a side effect (Brigo et al. 2014; Sharma et al. 2018). Therefore, animal models of HAL induction are suitable for assessing the motor functions of Parkinsonism (Waku et al. 2021).

In this study, administration of HAL to zebrafish artificially caused drug-induced Parkinsonism, and CBD was simultaneously administered to determine the improvement in motor dysfunction induced by D2 receptor inhibitory action. Zebrafish were used in this study because they are easy to handle, easy to observe due its transparency, economical, and lay many eggs simultaneously; moreover, their embryos develop quickly (Makhija and Jagtap 2014; Vaz et al. 2018). For verification of motor dysfunction, the amount of exercise based on the total distance traveled, along with the behavior pattern peculiar to zebrafish in response to light stimulation, were measured for a more comprehensive evaluation. In addition, an anti-Parkinsonian drug (Ropinirole) having a D2 receptor activating effect was administered separately, and the superiority or inferiority of improvement in comparison to that induced by CBD was verified.

## Methods

### Fish husbandry

The experiments were performed according to the Guide for the Care and Use of Laboratory Animals published by the US National Institutes of Health (NIH publication 85–23, revised 1996) and in accordance with the zebrafish experimental guide. The Experimental Animal Committee of Tokyo Medical University approved all experiments (approval number: H30-0020, R1-0114, R2-0037). Healthy, adult zebrafish (*Danio rerio*; purchased from Kamihata Fish Industries Ltd., Tokyo, Japan) were raised in aerated breeding units at a density of 2–3 fish per liter in dechlorinated municipal breeding water supplied by a recirculating water system and fed flake food twice daily. Fish were housed under the following conditions: pH, 7.5–8.0; conductivity, 300–500 μS/cm; temperature, 26–28 °C; and provision of light (8 AM to 10 PM) for 14 h and 10 h of darkness. To produce embryos, male and female zebrafish were paired in the evening, and fertilized embryos were collected and placed in petri dishes with fresh water. Embryos were transferred to a 28°C incubator on a 14:10 h light:dark cycle until 6 days post fertilization (6 dpf). Exposed embryos were screened to assess overall health, and dead embryos were removed on a daily basis.

### Drugs

HAL (Sigma-Aldrich, St. Louis, MO) was diluted with 0.05% dimethyl sulfoxide (DMSO; Dojindo Laboratories, Japan) in sterile saline (vehicle). The drug is a traditional antipsychotic agent used primarily to treat schizophrenia and other psychoses (Gomes et al. 2013; Vaz et al. 2018; Magno et al. 2015; Bruni et al. 2016) by relieving the symptoms of delusions and hallucinations commonly associated with schizophrenia. Haloperidol competitively blocks post-synaptic dopamine D2 receptors, eliminating dopamine neurotransmission while partially inhibiting 5-hydroxy-tryptamine (5-HT_2_) and α1-receptors. However, there is negligible activation of dopamine D1-receptors (Seibt et al. 2010).

CBD (Cayman Chemical, Ann Arbor, MI) was diluted with 0.05% methanol and sterile saline. CBD, one of the major compounds present in the marijuana (*C. sativa*) plant, has some medicinal properties; however, its mechanism is not well known (Andreza et al. 2016; Jeong et al. 2019).

Ropinirole hydrochloride (ROP; KYOWA Pharmaceutical Industry Co., Ltd, Osaka, Japan) was diluted with 0.05% dimethyl sulfoxide (DMSO; Dojindo Laboratories, Kumamoto, Japan) and sterile saline. The drug is a novel non-ergoline dopamine agonist, has selective affinity for dopamine D2 receptors, and is indicated for the treatment of early and advanced Parkinson’s disease (Pahwa et al. 2004).

### Light and dark test

Previous studies have evaluated locomotion with light stimuli using zebrafish (Magno et al. 2015; Irons et al. 2013; Connors et al. 2014; Liu et al. 2017). During evaluation of abnormal behavior, more comprehensive insights can be gained, not only by determining the levels of activity, but also via assessment of normal behavioral patterns and changes in these patterns attributable to drug effects. Considering that zebrafish display higher locomotor activity in the dark than in the light, the light and dark test is often used to analyze changes in fish locomotor activity and behavioral patterns because of drug effects (Basnet et al. 2019; de Esch et al. 2012). Evaluations based exclusively on the parameters of distance, speed, and duration of movement provide an insufficient assessment of drug effects; thus, we also compared behavioral patterns using the repeated light and dark test.

### Experimental procedure

Zebrafish larvae (6 dpf) were maintained in 96-well microtiter plates (one larva/well) (IWAKI Co. Ltd. Tokyo, Japan) filled with 300 μL breeding water per well. Locomotor activity was assessed after HAL (10 mg/L) induction, and CBD and ROP at 0.5, 1, 5, and 10 mg/L (16 larvae/group) were added directly into the water in the well. Simultaneous HAL+CBD treatments of 10 + 0.5, 10 + 1, 10 + 5, 10 + 10 mg/L (16 larvae/group) and HAL + ROP treatments of 10 + 0.5, 10 + 1, 10 + 5, 10 + 10 mg/L (16 larvae/group) were also assessed in trial 1 (*n*=192) (Gamelin et al. 2020). Furthermore, we also assessed the only CBD treatments of 1, 5, 10 mg/L (16 larvae/group) and the only ROP treatments of 1, 5, 10 mg/L (16 larvae/group) in trial 2 (*n*=144) (Oomen et al. 2018). The drug concentrations were determined in advance based on previous studies (Hasumi et al. 2020; Ellis et al. 2018).

Once the final concentration of the drug and the larvae were placed in the well, the distance traveled and the motion measurement at the time of illumination with bright (i.e., light-on periods) or infrared (i.e., darkness or light-off periods) light exposure by repeated light stimulation were examined until the end without replacing the larvae, water, and chemicals.

The light intensity used during the light-on period was 5000 lx, whereas under infrared illumination, the intensity was 0 lx. Fish were subjected to alternating (six times) light and dark exposure at 5-min intervals to determine differences in locomotor activities and response (Hasumi et al. 2020; Basnet et al. 2019; Yang et al. 2017; Maeda et al. 2021a; Maeda et al. 2021b). Drug effects were measured for 1 h using Danio Vision XT and Ethovision XT 11.5 (Noldus Information Technology, Wageningen, The Netherlands) (Maeda et al. 2018). Danio Vision is a high-throughput system that facilitates analysis of the behavioral responses of treated zebrafish and can simultaneously track up to 96 individuals (Maeda et al. 2021a) using the associated software. We examined the total distance traveled and movement response to the light and dark test. To measure spontaneous motion, by using a tracking system, the movement distance of the larvae in each light and dark time zone was tracked and the distance traveled in all time zones was summed.

Regarding the duration of drug treatment, the acclimation time was set at 30 min (Hasumi et al. 2020), and was followed by a total of six times as one set of light and dark times at 5-min intervals. Therefore, the total exposure time was 90 min. According to previous studies, the concentration of methanol, which is the medium of CBD, is 0.05% (Hasumi et al. 2020). However, this concentration has no effect in research (Hasumi et al. 2020).

### Statistical analysis

One-way analysis of variance (ANOVA) was used for statistical comparisons of the recorded observational data, followed by pairwise post-hoc comparisons using the Tukey–Kramer test. The data were analyzed using the Tukey–Kramer at a confidence level of 95%.

Data are presented as means ± standard errors of the mean (SEMs) of at least three independent experiments. Statistical analysis was performed in GraphPad Prism 6 for Windows version 6.05 (GraphPad Software, San Diego, CA, USA).

## Results

One-way ANOVA indicated that there were significant effects on total distance traveled (F=6.098, *p*<0.0001) in trial 1 (*n*=192), and there were also significant effects on total distance traveled (F=22.03, *p*<0.0001) in trial 2 (*n*=144).

### HAL-induced motor-dysfunction animal model (HAL 10 mg/L)

**Fig. 1.**
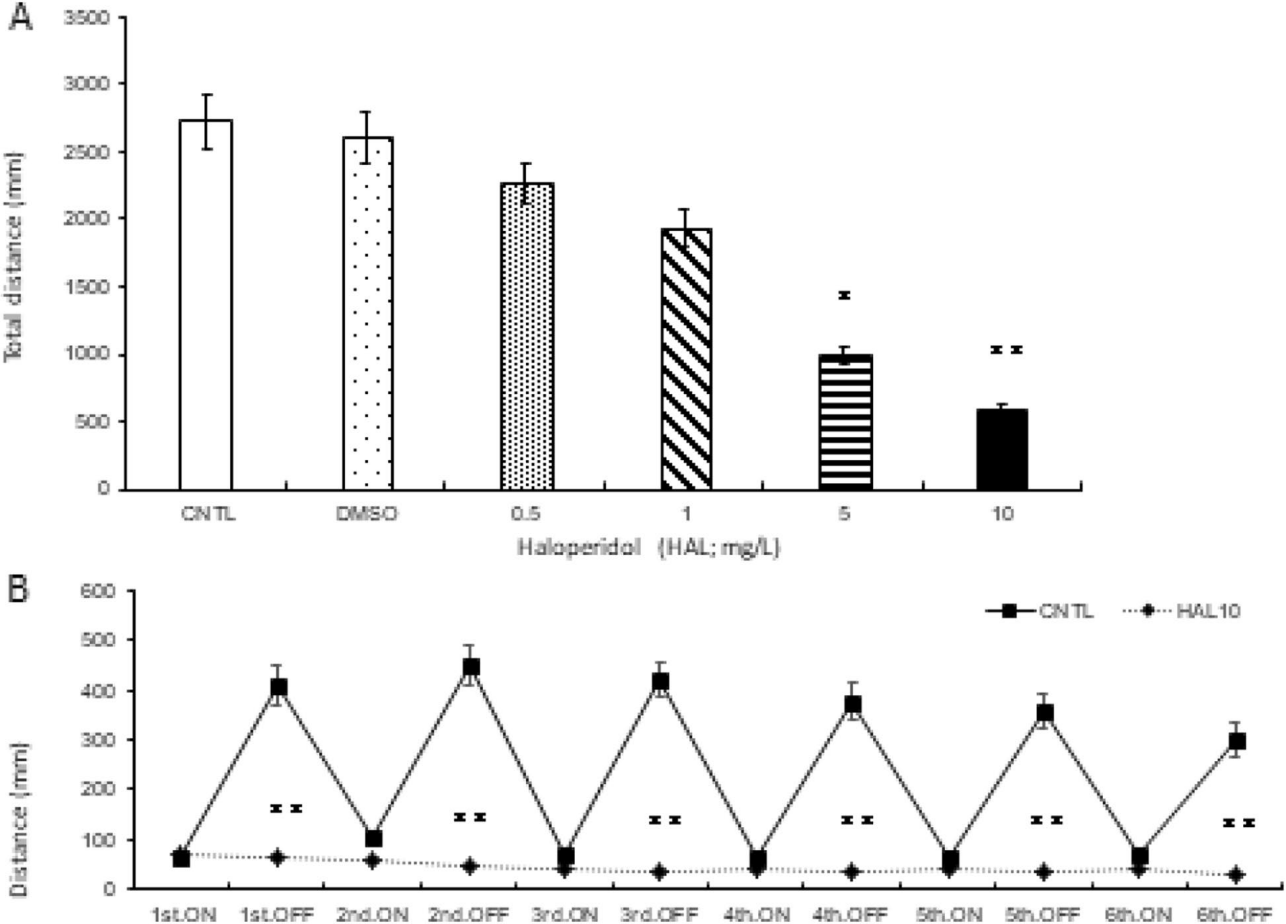
HAL-induced Parkinsonism zebrafish model. Comparison between the CNTL and HAL-treated groups at different doses (0.5, 1, 5, 10 mg/L). HAL at 10 mg/L exerted the strongest significant decrease in movement compared with the CNTL (**a**). The light (ON) /dark (OFF) -stimulation response of the HAL 10 mg/L-treated group was significantly reduced during all dark conditions (first to sixth) compared to that of the CNTL (**b**). CNTL: Control, DMSO: dimethyl sulfoxide 0.05%, HAL10: Haloperidol 10 mg/L, ON: light-on period, OFF: light-off period. Tukey-Kramer test; *p < 0.05, **p <0.01. All values are presented as means ± standard errors of the mean (SEMs) of at least three independent experiments (*n*=96)

First, we compared the total distance traveled between the control and HAL-treated groups at different doses (0.5, 1, 5, 10 mg/L). The HAL-treated group displayed the strongest effect at 10 mg/L (Fig. 1a). We also compared the light (ON)/dark (OFF)-stimulation response between the control and HAL (10 mg/L)-treated groups to verify whether 10 mg/L of HAL was adequate to reproduce the HAL-induced motor-dysfunction animal model. Zebrafish larvae normally exhibit hyperactivity in the dark and hypoactivity in light. We rotated between light and dark conditions every 5 min in 1-h trials. In the dark, control locomotor activity increased sharply, whereas light resulted in decreased activity. Comparing the control and HAL-10 mg/L groups, no significant difference was observed during lights ON, but the reactivity during lights OFF from the first to the sixth iteration was significantly reduced (*p*<0.01; Fig. 1b). Hence, 10 mg/L of HAL clearly decreased both activity and stimulus response, compared to the control group, and caused motor dysfunction-like symptoms.

### Effect of CBD on the motor-dysfunction animal model

**Fig. 2.**
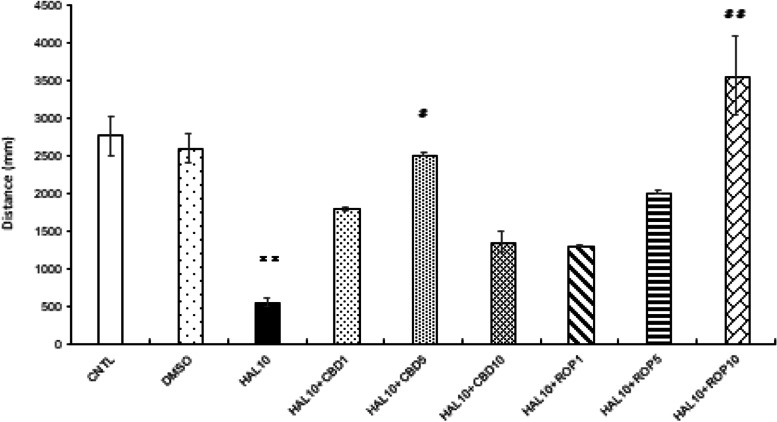
The effect of CBD and ROP on activity of zebrafish Parkinsonism model. Addition of 1, 5, 10 mg/L of CBD to 10 mg/L of HAL clearly improved the distance traveled at 5 mg/L of CBD compared to 10 mg/L of HAL. When ROP was added at 1, 5, 10 mg/L to HAL 10 mg/L, the travel distance was clearly improved at ROP 10 mg/L compared with HAL 10 mg/L. In addition, there is no significant difference between the HAL10 + CBD1 (mg/L), HAL10 + CBD5 (mg/L), HAL10 + ROP1 (mg/L), HAL10 + ROP5 (mg/L) and HAL10 + ROP10 (mg/L) compared to the CNTL and DMSO groups. CNTL: Control, DMSO: dimethyl sulfoxide 0.05%, HAL10: Haloperidol 10 mg/L, CBD 1, 5, 10: Cannabidiol 1, 5, 10 mg/L, ROP 1, 5, 10: Ropinirole 1, 5, 10 mg/L. Tukey–Kramer test; CNTL vs **p <0.01, HAL10 vs #*p* <0.05, HAL10 vs ##*p* <0.01, ns; not significant. All values are presented as means ± SEMs (*n*=144)

When CBD was administered at 1, 5, and 10 mg/L to the motor-dysfunction animal group (HAL-10 mg/L), the distance traveled was clearly improved at CBD 5 mg/L compared to the motor-dysfunction animal group (*p*<0.05; Fig. 2). In addition, there was no significant difference between the HAL 10 mg/L + CBD 1 mg/L, HAL 10 mg/L + CBD 5 mg/L, HAL 10 mg/L + CBD 10 mg/L and the control and DMSO groups (Fig. 2).

**Fig. 3.**
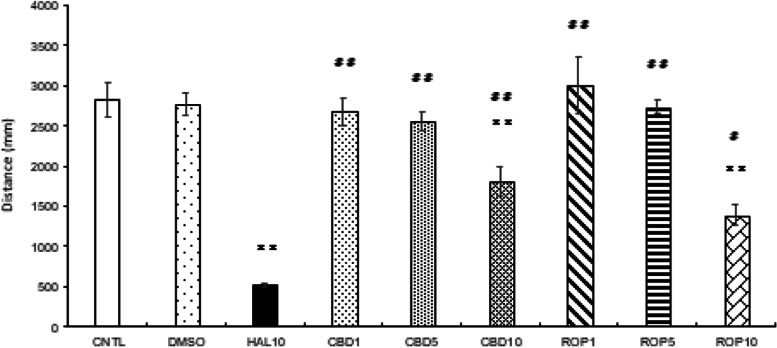
Effect of HAL 10 mg/L + CBD on light/dark-stimulation response. The distance traveled in the second light and sixth dark condition decreased in the HAL 10 +CBD 1 (mg/L) group compared with the CNTL (**a**). In the HAL10 +CBD5 group, the distance traveled in all light and dark conditions did not show a significant difference compared with the CNTL (**b**). In the HAL 10 mg/L + CBD 10 mg/L group, although the distance traveled in the first light condition increased, all dark conditions showed a significant decrease compared with the CNTL (**c**). In the HAL 10 mg/L + CBD 1 mg/L group, the response from the second to the fifth dark condition was significantly improved compared to the HAL 10 mg/L group (**d**). In the HAL 10 mg/L + CBD 5 mg/L group, the response was significantly enhanced in the fifth dark condition as compared with HAL 10 mg/L; however, the response was significantly improved in all dark conditions (**e**). In the HAL 10 mg/L + CBD 10 mg/L group, the response was enhanced only in the first and the third light conditions as compared with the HAL 10 mg/L group (**f**). CNTL: Control, HAL10: Haloperidol 10 mg/L, CBD 1, 5, 10: Cannabidiol 1, 5, 10 mg/L, ON: light-on period, OFF: light-off period. Tukey–Kramer test; *p <0.05, **p <0.01. All values are presented as means ± SEMs (*n*=128)

Activity under light stimulation decreased significantly in the HAL 10 mg/L + CBD 1 mg/L group compared to the control group in the second light condition (*p*<0.05) and the sixth dark condition (*p*<0.01; Fig. 3a). The HAL 10 mg/L + CBD 5 mg/L group showed no significant difference in all light and dark conditions compared to the control group (Fig. 3b). In the HAL 10 mg/L + CBD 10 mg/L group, the response was enhanced during the first light condition compared to the control group; however, the response significantly decreased during all dark periods (Fig. 3c).

Comparing HAL 10 mg/L and HAL 10 mg/L + CBD on the light/dark-stimulation experiment, the HAL 10 mg/L + CBD 1 mg/L group showed increased activity during the second to fifth dark periods, which was a significantly improved response compared to that of the motor-dysfunction animal group (Fig. 3d).

The HAL 10 mg/L + CBD 5 mg/L group had a significantly enhanced response during the sixth light condition compared to the motor-dysfunction animal group; however, the response was significantly improved during all dark conditions (Fig. 3e). In addition, the response of the HAL 10 mg/L + CBD 10 mg/L group was enhanced only in the first light condition (*p*<0.05) and in the third dark condition (*p*<0.01) compared to that of the motor-dysfunction animal group (Fig. 3f).

### The effect of ROP on the motor-dysfunction animal model

Similarly, when ROP was administered at 1, 5, and 10 mg/L to the motor-dysfunction animal group (HAL 10 mg/L), the distance traveled was clearly improved with ROP 10 mg/L compared to the motor-dysfunction animal group (*p*<0.01; Fig. 2). In addition, there was no significant difference between the HAL 10 mg/L + ROP 1 mg/L, HAL 10 mg/L + ROP 5 mg/L, and HAL 10 mg/L + ROP 10 mg/L groups compared to the control and DMSO groups (Fig. 2).

**Fig. 4.**
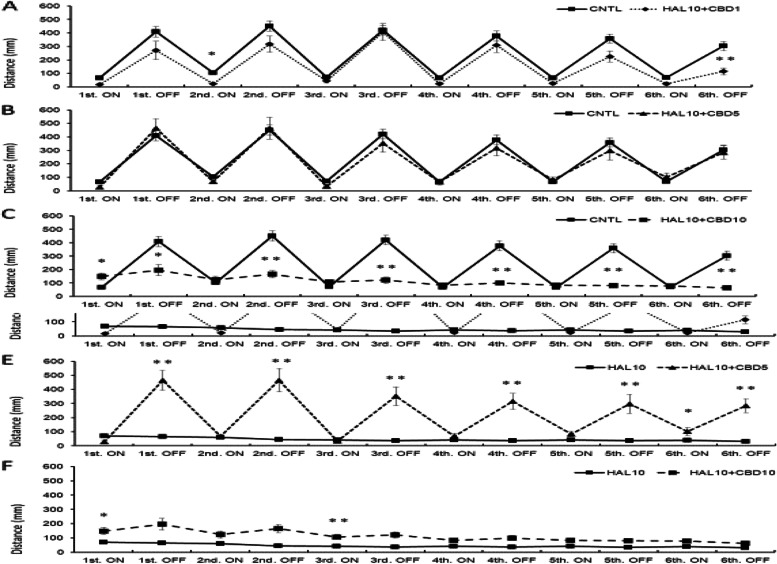
Effect of HAL 10 mg /L + ROP on light/dark-stimulatory response. In the HAL 10 mg/L + ROP 1 mg/L group, the response was enhanced in the first light condition compared to the CNTL; however, the response was significantly decreased in the second to sixth dark conditions (**a**). In the HAL 10 mg/L + ROP 5 mg/L group, the response significantly increased in all light conditions compared to the CNTL. However, the response significantly decreased in all dark conditions (**b**). In the HAL 10 mg/L + ROP 10 mg/L group, there was no significant difference in all dark conditions compared to the CNTL, but the response was enhanced from the first to fifth light conditions (**c**). The HAL 10 mg/L + ROP 1 mg/L group was significantly enhanced only in the first light condition compared with the HAL 10 mg/L group (**d**). The HAL 10 mg/L + ROP 5 mg/L group had a significantly enhanced response in all light conditions compared to the HAL 10 mg/L group (**e**). In the HAL 10 mg/L + ROP 10 mg/L group, the response was improved in all dark conditions, compared to the HAL 10 mg/L, whereas the response was enhanced in all light conditions (**f**). CNTL: Control, HAL 10: Haloperidol 10 mg/L, ROP 1, 5, 10: Ropinirole 1, 5, 10 mg/L, ON: light-on period, OFF: light-off period. Tukey–Kramer test; *p <0.05, **p <0.01 from the CNTL vs HAL 10 + ROP groups. All values are presented as means ± SEMs (*n*=128)

Although the response of the HAL 10 mg/L + ROP 1 mg/L group was enhanced compared with that of the control group in the first light period (*p*<0.05), the responses were significantly decreased during the second to sixth dark periods (*p*<0.01; Fig. 4a).

In the HAL 10 mg/L + ROP 5 mg/L group, the response was significantly enhanced in all lights-ON conditions compared to the control group. However, the response was significantly decreased in all lights-OFF conditions (Fig. 4b). In the HAL 10 mg/L + ROP 10 mg/L group, there was no significant difference in all dark conditions compared to the control group; however, the response was enhanced during the first to fifth light conditions (*p*<0.01; Fig. 4c). Comparison of the effects of the HAL 10 mg/L and HAL 10 mg/L + ROP groups on the light/dark-stimulatory response revealed that the effects of the HAL 10 mg/L + ROP 1 mg/L group were significantly enhanced only during the first light condition compared with those of the motor-dysfunction animal group (*p*<0.05; Fig. 4d). The HAL 10 mg/L + ROP 5 mg/L group had a significantly enhanced response in all lights-ON periods compared to the motor-dysfunction animal group (*p*<0.01; Fig. 4e). In the HAL 10 mg/L + ROP 10 mg/L group, the response was improved during all dark periods compared to that of the motor-dysfunction animal group; however, the response was enhanced in all light periods (*p*<0.01; Fig. 4f).

### Effect of CBD on normal animal model compared to the motor-dysfunction animal model

**Fig. 5.**
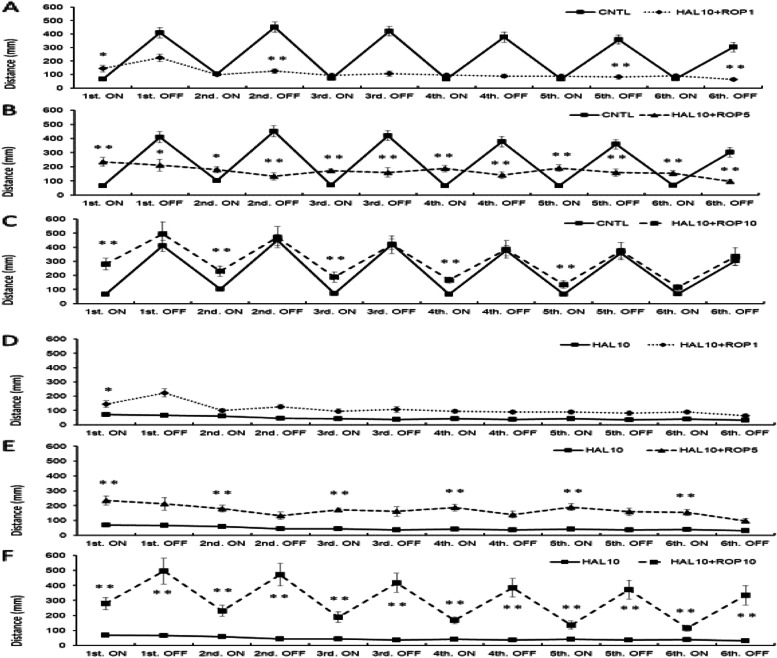
The effect of CBD and ROP on activity of zebrafish normal model. Addition of 1, 5, 10 mg/L of CBD and ROP to the normal model clearly were different from HAL 10 mg/L. Especially, the travel distances at both CBD 10 mg/L and ROP 10 mg/L were clearly different from the CNTL. CNTL: Control, DMSO: dimethyl sulfoxide 0.05%, HAL10: Haloperidol 10 mg/L, CBD 1, 5, 10: Cannabidiol 1, 5, 10 mg/L, ROP 1, 5, 10: Ropinirole 1, 5, 10 mg/L. Tukey–Kramer test; CNTL vs **p <0.01, HAL10 vs #*p* <0.05, HAL10 vs ##*p* <0.01, ns; not significant. All values are presented as means ± SEMs (*n*=144)

When CBD was administered at doses of 1, 5, and 10 mg/L to the normal animal group, the distance traveled clearly showed that all groups were different from the motor-dysfunction animal group (*p*<0.01; Fig. 5). In addition, there was a significant difference between the CBD 10 mg/L and the control group (*p*<0.01; Fig. 5).

**Fig. 6.**
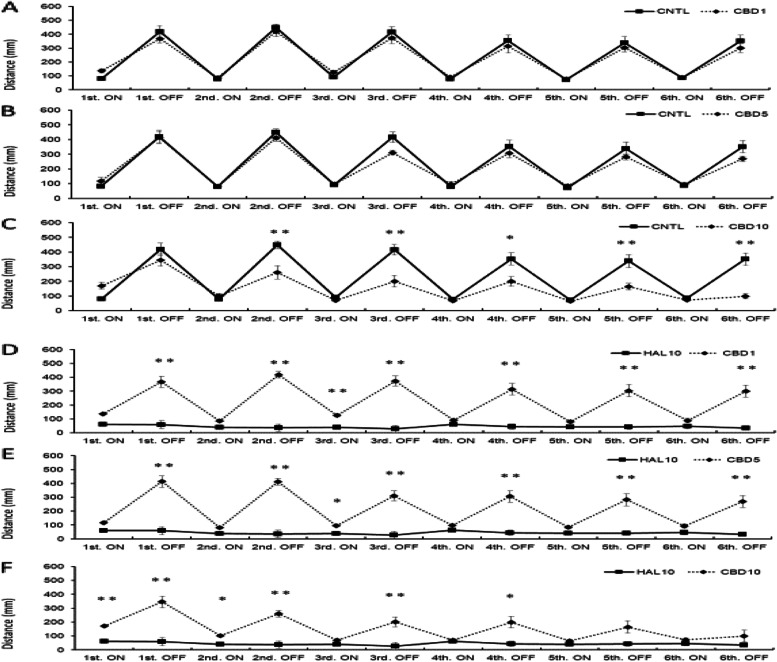
Effect of CBD on light/dark-stimulation response. There were no significant differences from CNTL and CBD 1 mg/L nor 5 mg/L (**a**, **b**). The distance traveled in the second, third, fourth, fifth, and sixth dark condition decreased in the CBD 10 mg/L group compared with the CNTL (**c**). In the CBD1 and CBD5 groups, the distance traveled in all dark conditions showed a significant difference compared with HAL 10 mg/L (**d**, **e**). In the CBD 10 mg/L group, although the distance traveled in the first and second light and dark conditions, the third and fourth dark conditions showed a significant difference compared with HAL 10 mg/L (**f**). CNTL: Control, HAL10: Haloperidol 10 mg/L, CBD 1, 5, 10: Cannabidiol 1, 5, 10 mg/L, ON: light-on period, OFF: light-off period. Tukey–Kramer test; *p <0.05, **p <0.01. All values are presented as means ± SEMs (*n*=128)

Activity under light/dark stimulation did not decrease significantly in the CBD 1 mg/L and CBD 5 mg/L groups compared to control (*p*<0.05, *p*<0.01; Fig. 6a, b); however, activity under dark conditions decreased significantly in the CBD 10 mg/L group compared to control (*p*<0.05, *p*<0.01; Fig. 6c).

**Fig. 7.**
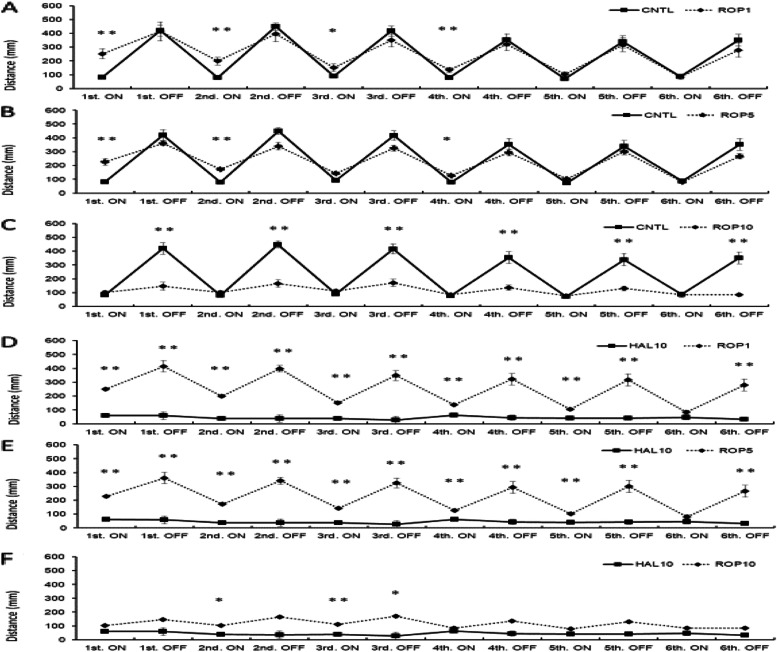
Effect of ROP on light/dark-stimulatory response. In the ROP 1 mg/L and 5 mg/L groups, the response was enhanced in the first, second, third, and fourth light condition compared to the CNTL (**a**, **b**); however, all light and dark conditions except sixth light were significantly different from HAL 10 mg/L (**d**, **e**). In the ROP 10 mg/L group, the response was significantly decreased in all dark conditions compared to the CNTL (**c**); however, the second and third light/dark conditions were significantly different from HAL 10 mg/L (**f**). CNTL: Control, HAL 10: Haloperidol 10 mg/L, ROP 1, 5, 10: Ropinirole 1, 5, 10 mg/L, ON: light-on period, OFF: light-off period. Tukey–Kramer test; *p <0.05, **p <0.01 from the CNTL vs the HAL 10 + ROP groups. All values are presented as means ± SEMs (n

Comparing HAL 10 mg/L and CBD on the light/dark-stimulation experiment, the CBD 1 mg/L and CBD 5 mg/L groups showed significant differences especially under dark stimulation (*p*<0.05, *p*<0.01; Fig. 7a, B). The CBD 10 mg/L group had significantly different responses during the first through fourth light/dark conditions compared to those of the motor-dysfunction animal group (*p*<0.05, *p*<0.01; Fig. 7c).

### The effect of ROP on normal animal model compared to the motor-dysfunction animal model

When ROP was administered at 1, 5, and 10 mg/L to the normal animal group, the distance traveled clearly showed that all groups were different from the motor-dysfunction animal group (*p*<0.05, *p*<0.01; Fig. 5). In addition, there was a significant difference between ROP 10 mg/L compared to the control (*p*<0.01; Fig. 5).

Comparing the ROP 1 mg/L and 5 mg/L groups to the normal animal group, the distance traveled under the first through fourth light periods was clearly enhanced compared to CNTL (*p*<0.05, *p*<0.01; Fig. 6a, b); however, the response of the ROP 10 mg/L group decreased in all dark conditions compared with that of the CNTL (*p*<0.01; Fig. 6c). In addition, there was a significant difference between ROP 10 mg/L compared to the motor-dysfunction animal group except for the sixth light period (Fig. 7d, e); however, the response of the ROP 10 mg/L group was different only in the second light and third light/dark conditions compared with that of the motor-dysfunction animal group (*p*<0.05, *p*<0.01; Fig. 7f).

## Discussion

The purpose of this study is to examine whether CBD has an ameliorating effect on HAL-induced motor dysfunction, using zebrafish, and its effect was compared to that of an anti-PD drug. Before examining the effect of CBD, we needed a HAL-induced motor-dysfunction animal model; hence, we started by trying to reproduce one.

Normal zebrafish display low activity levels during light periods, while in the dark, they are hyperactive (Maeda et al. 2021b). This property can be used to investigate the effects of each drug on dopamine receptors in zebrafish. Therefore, when HAL, CBD, and ROP were administered individually to zebrafish, a significant decrease in activity was observed starting from 5–10 mg/L for HAL, and a similar decrease was observed in response to both the light and dark conditions at the 10-mg/L dose. Therefore, HAL 10 mg/L was used as the standard for movement disorders. There was no significant change in ROP and CBD at the same concentration compared to the normal group, but as with the concentration of 10 mg/L, activity decreased as the concentration was as high as that of HAL. However, for repetitive photostimuli, a unique pattern was confirmed: CBD decreased reactivity from the 10 mg/L dark band, while ROP increased at light for 1–5 mg/L, but conversely decreased at dark at 10 mg/L. Thus, it is important to understand the characteristics of the behavior pattern of each drug in light and dark. Confirming abnormal patterns of activity (hypoactivity and hyperactivity) will set a standard for further investigation of the synergistic effects between drugs. It can also guide future research on developing a zebrafish model for studying movement disorders.

A comparison between the control (or normal) and HAL 10 mg/L groups revealed a significant difference in the total distance traveled and activity during lights-OFF; however, there was no significant difference during lights-ON. In the group with D2 receptor inhibition, decreased response during lights-ON was not different from that in the normal group. However, motor dysfunction was confirmed, in which the reactivity was significantly reduced even during the dark periods.

This experiment shows that a HAL-induced motor-dysfunction animal model can be reproduced by administration of HAL 10 mg/L and the total distance traveled and the light/dark-stimulation response could be measured. Next, as a result of administering CBD and ROP of each concentration group to this Parkinsonism model, the CBD 5 mg/L and ROP 10 mg/L groups showed a significant improvement in the total distance traveled compared to the Parkinsonism model group, respectively. From this result alone, the improvement in momentum in both cases could be seen; however, the results were significantly different between the CBD 5 mg/L and ROP 10 mg/L groups, in terms of the light-stimulation response. In an experiment comparing the normal and HAL 10 mg/L groups, activity was significantly different during dark periods, but not during light period. In experiments using CBD, administration of CBD 5 mg/L to the HAL-induced motor-dysfunction model group showed no significant difference during the light periods. However, activity was improved during dark periods. In experiments using ROP, administration of ROP 10 mg/L improved activity in the dark, but the fact that activity was significantly higher in the light was clearly an abnormal increase compared to the normal group. Therefore, examination of the total distance traveled and light-stimulation response showed that CBD 5 mg/L has a higher efficacy on the HAL-induced motor-dysfunction model group than ROP 10 mg/L. Specifically, at 5 mg/L, the ameliorating effect of CBD required approximately half of the HAL concentration (10 mg/L) and was more effective than the other CBD concentration groups (1 and 10 mg/L). Furthermore, although administration of ROP showed a concentration-dependent improvement in the HAL-induced motor-dysfunction model group, CBD 5 mg/L made the zebrafish behave more normally than the various ROP concentrations (1, 5, 10 mg/L). Therefore, the movement disorder caused by the pharmacological effect of HAL (at 10 mg/L) could be improved most by half the concentration of CBD (5 mg/L), and the D2 activation effect exerted by CBD was stronger than the D2 inhibitory effect exerted by HAL.

The effect of CBD 5 mg/L was higher than that of ROP at the same concentration (5 mg/L). ROP was more effective at 5 mg/L and less effective at 10 mg/L. Hence, CBD might be better than ROP and may show efficacy as an anti-PD drug. 5-HT1A receptors are deeply involved in serotonin regulation but are also implicated in the regulation of dopamine levels in the brain (Hu 2016; Di Giovanni et al. 2008). In fact, some reports have suggested that cannabis improves Parkinsonism (Venderová et al. 2004); hence, a future pharmacological challenge is directly influencing motor dysfunction promoted by drug-induced D2 receptor blockade. It is necessary to verify whether the mediation effect of 5-HT1A receptor activation of CBD has a higher improvement effect than that of dopamine D2 receptor activation. Concerning drug toxicity, morphological changes or toxicity due to HAL, CBD, or ROP have not been identified with respect to abnormalities in activity with high doses.

Similarly, using zebrafish, many research papers on drug-induced catalepsy symptoms have evaluated behavioral actions based on decreasing duration of movement due to D2 receptor blocking, including the delay in start time and the total distance travelled (Flinn et al. 2008; Stewart and Kalueff 2014; Tierney 2011). Therefore, in this study, not only the amount of exercise (total distance traveled), but also the normal and abnormal movement patterns, were compared to evaluate the activity and motor function as a whole.

The behavioral characteristics of zebrafish are important, and in normal larvae, their activity decreases under bright light stimulation and increases in dark conditions. This is a normal behavioral pattern and an indicator of the reaction (Hasumi et al. 2020; de Esch et al. 2012). The total distance traveled within the measurement time can determine the strength of the overall activity, but not the abnormality of the behavior pattern. For example, if the total distance traveled under the influence of drug A is the same as that traveled by an untreated zebrafish, but the total distance traveled under the influence of drug B is significantly reduced, it can be considered that motor function is affected by drug B but not by drug A. However, when comparing behavioral patterns, if remarkable abnormal behavior is always observed with drug A, it cannot be stated that drug A has no effect on motor function. Furthermore, to date, it is believed that cannabinoids and dopamine are closely related (Covey et al. 2017); however, the degree of recovery of drug-induced Parkinsonism-like symptoms by CBD to a state close to normal is yet to be determined. In this study, we were able to verify the effect of CBD more specifically by pursuing the concentration ratio at which HAL and CBD become antagonistic and by comparing them with anti-PD drugs (ROP) of the same concentration.

There have been many pharmacological studies on dopamine in zebrafish, and it has been found that motor dysfunction due to dopamine receptor inhibitory action is similar to that of humans (Vaz et al. 2018). In addition, there are comparative studies of zebrafish models and rodent models in Parkinson's disease studies (Makhija and Jagtap 2014), but the zebrafish model as a whole has not yet been established. Interactions between dopamine receptors and cannabinoids in rodents have also been reported in several papers (Andreza et al. 2016; Gomes et al. 2013).Although it has been elucidated that CBD's neuroprotective effect (Patricio et al. 2020) and dopamine receptor activation via serotonin receptors contribute to improved motor function (Espejo-Porras et al. 2013; Andreza et al. 2016; Gomes et al. 2013), it is necessary to verify the same in zebrafish in the future.

## Conclusion

Clinical application-focused research on CBD is currently progressing dramatically, and the results of this study indicate that CBD at approximately half the concentration of haloperidol, which has a D2 blocking effect, ameliorates the symptoms. Our findings are only the tip of the iceberg. However, the finding that CBD, which has no direct D2 receptor action, is effective against Parkinsonism-like symptoms induced by inhibition of D2 receptor action will change our considerations concerning cannabinoids in the future.

To date, CBD research has been conducted to determine its antiepileptic action or antipsychotic side effects (Devinsky et al. 2017; Andreza et al. 2016); however, CBD is also expected to have a therapeutic effect on neurodegenerative diseases, such as Parkinson’s and Alzheimer’s diseases, due to receptor blockade and the neuroprotective effect of anti-inflammation and anti-oxidation (Patricio et al. 2020; Cooray et al. 2020; Crippa et al. 2018). However, the applied research for neuromotor and neurodegenerative diseases by CBD for the purpose of full-scale treatment has not yet reached the scale of clinical trials. Hence, this research is at a stage where further basic experiments need to be conducted in the future. This study provides basic research data on the improvement effect of CBD on dopamine receptor-induced motility disorder in the striatum.

## Data Availability

Data and materials are available upon request to the corresponding author.

## Abbreviations

CBD: Cannabidiol
CNTL: Control
DMSO: Dimethyl sulfoxide
HAL: Haloperidol
ROP: Ropinirole
ANOVA: Analysis of variance
